# Effect of LSVT on Lexical Tone in Speakers with Parkinson's Disease

**DOI:** 10.4061/2011/897494

**Published:** 2011-08-23

**Authors:** Tara L. Whitehill, Lorinda Kwan, Flora P.-H. Lee, Mia M.-N. Chow

**Affiliations:** Division of Speech and Hearing Sciences, The University of Hong Kong, Pokfulam, Hong Kong

## Abstract

Lee Silverman Voice Treatment (LSVT) has well-documented treatment efficacy for individuals with hypokinetic dysarthria associated with Parkinson's disease (PD). Positive changes have been noted after treatment not only for vocal loudness but also for many other speech dimensions, including intonation (monotonicity). There have been few studies investigating the effect of LSVT on lexical tone which, like intonation, is controlled by variations in fundamental frequency. This study involved 12 Cantonese speakers with idiopathic PD who were enrolled in a standard LVST treatment protocol. Speech data were collected 3-4 days before treatment and 1 day after treatment. A wide variety of perceptual and acoustic variables were analyzed. The results showed significant improvements in loudness and intonation after treatment, but no significant changes in lexical tone. These results have theoretical implications for the relationship between tone and intonation and for models of the physiological control of fundamental frequency.

## 1. Effect of LSVT on Lexical Tone in Speakers with Parkinson's Disease

Lee Silverman Voice Treatment (LSVT), which focuses on increasing vocal loudness, was developed for the treatment of voice and speech impairment in individuals with Parkinson's disease (PD). The treatment protocol involves intensive treatment delivery (a one-hour session, four days a week for four weeks) and has been fully described elsewhere [1]. LSVT has well-documented treatment efficacy (e.g., [2, 3]). Positive changes have been noted not only for vocal loudness but also for many other speech dimensions, including intonation [1, 2]. Monotonicity, a disruption in intonation, is considered one of the hallmarks of hypokinetic dysarthria [4]. 

An estimated 60–70% of the world's languages are tonal [5]; that is, where words of different meaning can be marked by variations in tone alone. Whereas intonation is associated with variation in fundamental frequency at the phrasal level, lexical tone is associated with variation in fundamental frequency at the syllable level. Cantonese (Chinese) has six contrastive tones, which vary according to pitch height and pitch contour [6]. Using the numerical system developed by Chao [7], where the first number represents the beginning level of the tone and the second number indicates the finishing level of the tone, the six lexical tones of Cantonese are 55 (high level), 35 (high rising), 33 (mid level), 21 (low falling), 23 (low rising), and 22 (low level). 

 There have been few studies of hypokinetic dysarthria in speakers of lexical tonal languages. Cantonese speakers with PD have been found to demonstrate similar characteristics, in terms of disrupted dimensions of speech, as English and Japanese speakers with the disease [8]. Lexical tone was found to be relatively unimpaired, in contrast to “monotone,” which was one of the most severely affected dimensions of speech in this group of Cantonese speakers [8]. This contrast in findings for lexical tone and intonation suggests possible differential control for these two functions, a hypothesis previously advanced by Vance [9]. A pilot study of four Cantonese speakers with PD following a treatment program based on LSVT showed improvements in intonation but little change in lexical tone impairment [10]. The authors noted that the four speakers had relatively intact lexical tone production before treatment. The current study is an extension, employing a larger number of participants with PD, and treatment by clinicians certified in LSVT. 

The aim of the current study was to determine whether LSVT, which has well-documented success in improving monotone speech (as well as other disordered speech dimensions) in individuals with PD, would have a similar positive impact on lexical tone errors.

## 2. Method

### 2.1. Participants

 The speaker participants were 12 Cantonese speakers with idiopathic PD (5 males, 7 females; age range 56–78 years). All these participants received regular medication for PD that was unchanged throughout the course of the study, except for one participant whose medication was modified during the study. Improvements in speech due to dopamine-type medication are believed to be small and are highly varied across different individuals [11]; hence, this participant was still included in the study. All speakers had normal oral-peripheral structures and passed a hearing screening at 40 dBHL at 500, 1000, 2000, and 4000 Hz for the better ear. In addition, all passed a screening for aphasia and apraxia based on the Cantonese Aphasia Battery [12]. Number of years since diagnosis ranged from 4 to 23. The listener participants for most of the perceptual tasks were 12 speech-language pathology students (for the tone error identification task, described later, three experienced speech-language therapists served as listeners, because this task was conducted after the main experiment). Signed consent was obtained from all participants, and the project was approved by the appropriate Ethics Committee at the University of Hong Kong. 

### 2.2. Treatment

Treatment was provided by 12 qualified native Cantonese speech-language therapists who had recently completed an LSVT certification course. The standard LSVT treatment protocol was followed [13]. All 12 speakers completed 16 individual treatment sessions within four weeks.

### 2.3. Speech Materials and Data Collection

 The data reported here were collected three or four days before treatment and one day after treatment. All data were collected by investigators who were not involved in delivering the treatment. Recordings were made in a quiet room with background noise level of less than 43 dB. Speech samples were recorded using an Aardvark Direct Mix USB 3 Soundcard and Audacity 1.2.6. An AKG C 525 S or Shure SM48 low-noise unidirectional microphone was held at a mouth-to-microphone distance of 10 cm. A wide range of speech stimuli was employed; this study focuses only on a 30-second speech sample extracted from a standard reading passage (the Chinese “Barbra Streisand” passage [14]). All sentences and syllables were low-pass filtered (cutoff frequency 3000 Hz) using Praat, Version 5.1 [15]. The samples were randomized across speakers and time (pre versus posttreatment). The intensity of all speech samples (except samples for the perceptual rating of vocal loudness) was normalized using Praat, Version 5.1 [15] to eliminate the possible effect of loudness on perceptual judgments. A wide range of outcome measures were analyzed perceptually and acoustically, including measures relating to loudness, vocal quality, intonation, speech rate, and lexical tone; this study focuses only on the intonation and tone measures. The results for the other outcome measures are available elsewhere [16, 17].

### 2.4. Perceptual Analysis

The perceptual rating tasks were conducted individually in a sound-attenuated booth using Windows media player running on a Compaq Presario V3000 laptop and Sennheiser HD 212Pro headphones. The order of the rating tasks was randomized across listeners to control for order effects. Monotonicity was rated by the 12 student-listeners using a visual analogue scale (VAS). The left side of a 10 cm line was labeled “normal” and the right side “extremely monotone.” The listeners were asked to mark a cross on the line to represent their judgment of each speaker's monotonicity. 

 Lexical tone was analyzed using two tasks, tone transcription, and tone error identification. Data from one speaker were excluded due to presence of a dialect that affected tone. The first task used syllables extracted from the reading passage. Three tokens were included for each of the six tones. These single-word tokens were randomized across speakers and time. Each token was transcribed by the listeners by writing down the tone value (55, 35, 33, 21, 23, or 22). The second task involved extracting ten phrases from the reading paragraph, which totalled 65 syllables. Three tokens were included for each of the six tones. This task was conducted subsequent to the main experiment, due to poor intrarater reliability for the tone transcription task. The listeners for this task were three experienced speech-language therapists who were asked to listen to the sentences, follow the written text, and circle any syllable that they perceived to be in error.

### 2.5. Acoustic Analysis

 Standard deviation of fundamental frequency (SDFO) was used as the acoustic correlate for monotonicity. Mean F0 and SDFO values were calculated from the 30-second reading passage sample, using the autocorrelation algorithm in Praat, Version 5.1 [15]. The F0 range was set between 75–300 Hz for males and 100–500 Hz for females. The samples for two older female participants whose voices were low-pitched were analyzed using the male pitch ranges. In order to normalize the speech productions from two genders, the SDFO values originally measured in Hertz were converted into a logarithmic semitone (ST) scale [18, 19].

For lexical tone, fundamental frequency (FO) was measured for each extracted syllable (the same syllables used in the tone transcription task; three tokens for each tone). The voiced segment of each of the eighteen stimuli was identified auditorially, by listening to the signal, and visually, from a wideband spectrogram and an amplitude waveform display. The voiced segment was defined as the third cycle from the start to the third cycle from the end [20]. F0 was measured at five time points of this segment (i.e., 0%, 25%, 50%, 75%, and 100% of the total duration) and was calculated using the autocorrelation algorithm in Praat software, Version, 5.1 [15]. By averaging the F0 of all three tokens of each tone at each time point, the tone configuration of each participant was determined. The F0 values were then converted from the Hertz unit to semitones, in order to normalize the interspeaker differences for statistical analysis.

### 2.6. Reliability

Intrarater reliability for monotonicity (based on repeating the stimuli from two speakers) was 0.70 (Pearson's *r, P*  <  0.01); interrater reliability was 0.71 (ICC, 3, k). Intrarater reliability for tone transcription was 0.51 (Pearson's *r*, *P*  <  0.01) and interrater reliability was 0.91 (ICC, 3, k). Intrarater reliability for the tone error identification task was 0.96 (Pearson's *r*, *P*  <  0.01) and interrater reliability was 0.70 (ICC, 3, k). 

Inter- and intrarater reliabilities for the acoustic measures were calculated by repeating the analysis for two speakers by the investigator and a second rater. Intrarater reliability was 0.99 (Pearson's *r*, *P*  <  0.05) and interrater reliability was 0.95 (Pearson's *r*, *P*  <  0.05) for both acoustic measures.

### 2.7. Statistical Analysis

To evaluate the efficacy of LSVT, pre and posttreatment changes across speech dimensions were calculated using both descriptive and inferential statistics. A repeated measures multivariate analysis of variance (MANOVA) was carried out for analysis of the perceptual measures of voice quality, vocal loudness, intonation, and rate, while a two-way, repeated-measures ANOVA was carried out for analysis of lexical tone accuracy (the error identification task). A repeated measure multivariate analysis of variance (MANOVA) was also computed for the four acoustic variables, with a separate three-way ANOVA for lexical tone.

## 3. Results

### 3.1. Perceptual Measures

The mean perceptual rating for the speech dimension “monotone” on the 10 cm VAS scale, where a higher number indicated more severely monotone speech, was 3.13 (SD  =  1.84) before therapy and 2.34 (SD  =  1.72) after therapy. This decrease in monotonicity was statistically significant, *F*(1, 11) = 19.97,*P*  <  0.001.

The mean accuracy of lexical tone, based on transcription, was 54.02 (SD  =  0.16) before therapy and 56.62 (SD  =  0.17) after therapy. Statistical analysis was not conducted for this measure because of the low intrarater reliability of the task. The mean accuracy of lexical tone based on identifying inaccurate tones was 97.72 (SD  =  0.03) before treatment and 97.64 (SD  =  0.03) after treatment. This difference was not statistically significant, *F*  =  0.13, *P*  =  0.724.

### 3.2. Acoustic Measures

**Figure 1:**
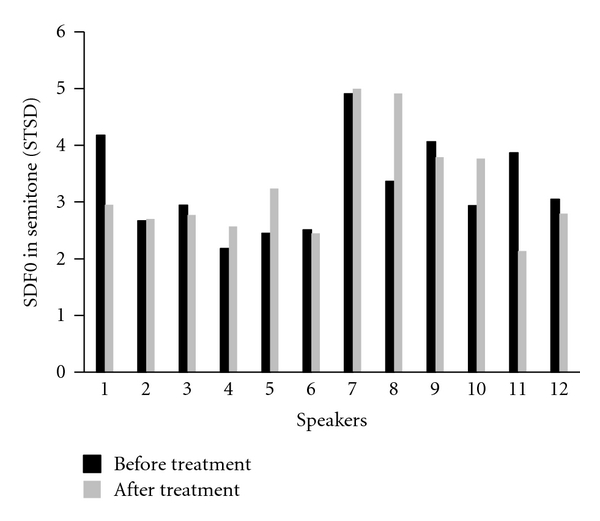
Standard deviation of fundamental frequency (SDF0), in semitones, for individual participants.

Mean SDF0, in semitones (STSD), was 3.259 (SD  =  0.83) before treatment and 3.256 (SD  =  0.93) after treatment. There was no significant treatment effect, *F*(1,11)  <  0.001, *P*  =  0.9. However, examination of individual data revealed an increase in SDST for six of the twelve subjects. Two participants had a noticeable decrease in SDST after treatment (S1 and S11). The results for individual speakers are shown in Figure 1. 

A three-way repeated measures ANOVA was used to analyze the lexical tone data with the within-group factors of time (pre and posttreatment), tone (tone 55, tone 25, tone 33, tone 21, tone 23, and tone 22), and time points (0%, 25%, 50%, 75%, and 100%). Significant main effects were observed for time, *F*(1,11)  =  7.80, *P*  <  0.05, tone, *F*(5, 55)  =  17.95, *P*  <  0.001 and time point, *F*(4, 44)  =  23.28, *P*  <  0.001. The main effect of time indicated that overall mean F0 was higher after treatment than before treatment. A statistically significant difference was also indicated in the interaction of tone and time point, *F*(22, 220)  =  14.95, *P*  <  0.001. This confirmed that different tones have different frequencies at different time points and it is not related to any changes in treatment. No significant changes in time-tone, time-time point and time-time point-tone interactions were shown. This indicates that treatment effects were the same across all tones and all time points and implies that the F0 contour pattern of each tone had no significant statistical changes from before to after treatment.

The F0 patterns for all speakers were examined individually, in order to identify individual changes or patterns. For this analysis, F0 was not converted to semitones. Findings for the speakers with PD were compared with previously reported normative data for nonimpaired Cantonese speakers [21, 22]. All the male PD speakers generally showed similar F0 heights to nonimpaired male speakers in the pretreatment condition. After treatment, three of the male speakers showed an increase in F0 height, with two appearing above-normal values while there was no change in F0 height for the remaining two. For the female speakers, F0 heights were generally lower than the normative values and remained similar before and after treatment, although two of the female speakers exhibited an upward shift of F0 height across all six lexical tones and one showed a reduction in F0 height after treatment. 

**Figure 2:**
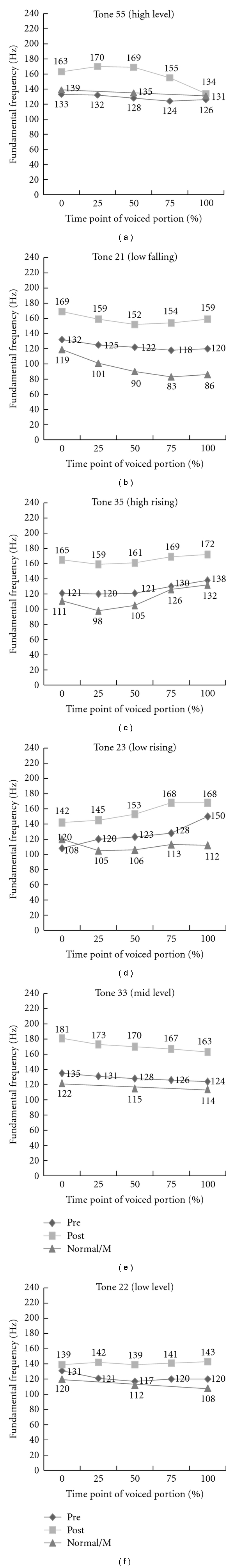
F0 pattern of six lexical tones produced by a 60-year-old male, WSH. The normative data are from Whitehill et al. [21, 22], cited in Whitehill and Wong [10].

**Figure 3:**
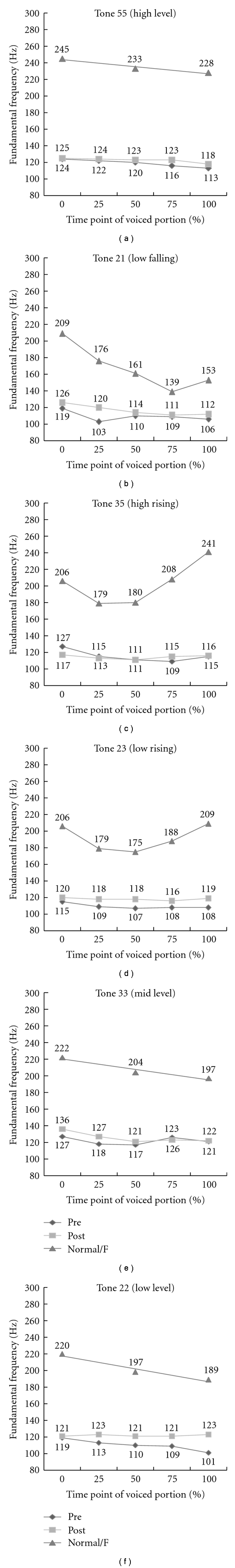
F0 pattern of six lexical tones produced by a 78-year-old female, HYH. The normative data are from Whitehill et al. [21, 22], cited in Whitehill and Wong [10].

**Figure 4:**
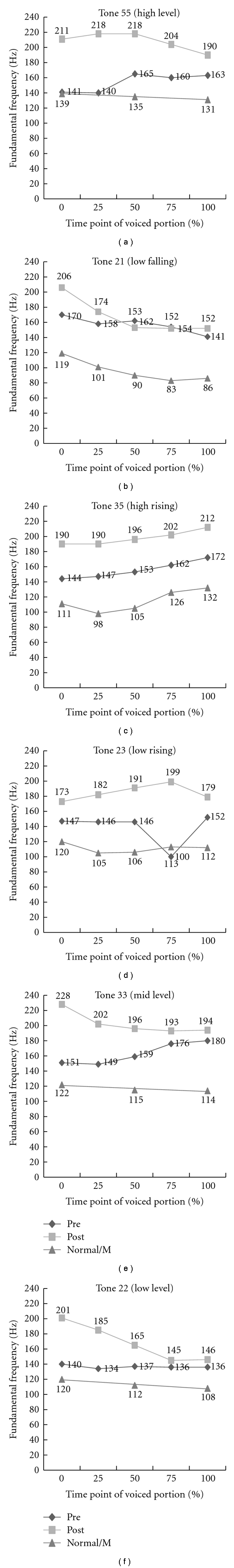
F0 pattern of six lexical tones produced by a 56-year-old male, CWY. The normative data are from Whitehill et al. [21, 22], cited in Whitehill and Wong [10].

Two of the twelve participants demonstrated similar F0 contour patterns before and after treatment to the nonimpaired speakers across all six tones.  Figure 2 shows one of these two speakers, WSH, who was considered to have normal lexical tone production both before and after treatment. The remaining speakers generally showed similar F0 patterns to the nonimpaired speakers for the three level tones (i.e., tones 55, 33 and 22) while the contour tones (i.e., tones 35, 21, and 23) were observed to be flattened. Six speakers demonstrated flattening of all contour tones in the pretreatment condition. Three of these speakers showed no change in the F0 pattern of these contour tones after treatment while another three showed improvement after treatment although not on all three contour tones.  Figure 3 shows the results for one speaker, HYH, whose F0 pattern of the contour tones remained unchanged after treatment. As can be seen, the F0 configurations across all six tones were at a similar F0 height level and showed a similar contour pattern before and after treatment.  Figure 4 shows a speaker, CWY, who had a normal tone contour pattern after treatment on one of the contour tones (tone 21). However, there were also some abnormal patterns observed (e.g., tone 23 and tone 33 before treatment and tone 22 after treatment). For four of the affected participants who were not yet mentioned, their lexical tone production had no clear pattern. That is, a normal F0 pattern might be found before treatment but an abnormal/flattened one after treatment, or vice versa. In summary, the qualitative analysis of lexical tone indicated that the most of the abnormal lexical tones produced by the participants with PD before treatment (i.e., the contour tones) remained flattened after the treatment.

### 3.3. Other Treatment Variables

Although not detailed here, significant group treatment effects were additionally found for the dimensions of excessive soft voice and excessive loud voice (perceptual variables) and for sound pressure level and mean fundamental frequency (acoustic variables; for details, see Chow [16]; Lee [17]). These results were generally consistent with previous reports for LSVT (e.g., Ramig et al. [23]) and show that the treatment provided was successful in terms of several outcomes measures traditionally targeted in this population.

## 4. Discussion

 LSVT has well-established efficacy for the treatment of speech disorders in individuals with hypokinetic dysarthria associated with PD. The results of this study showed that the treatment approach was also successful with this group of Cantonese speakers with PD, based on several traditional outcome measures, as noted above. This was consistent with the results of a previous small-scale study with Cantonese speakers [10]. The main focus of this study was on the effect of LSVT on lexical tone in PD speakers. We also examined treatment effects for intonation (monotone) since, like lexical tone, intonation is primarily controlled by variations in fundamental frequency. We employed both perceptual and acoustic measures, and undertook qualitative analysis of individual speakers as well as statistical analysis of group results. 

 For the disordered speech dimension of “monotone”, there was a significant improvement for the group, based on listeners' perceptual ratings. That is, the speakers were less monotone after treatment. In contrast with the perceptual findings, statistical analysis of the group results for the acoustic correlate of monotonicity, SDST, showed no significant difference before and after treatment for the group. This was inconsistent with previous reports of LSVT treatment in both English speakers (e.g., Ramig et al. [23]) and Cantonese speakers [10]). However, examination of individual results showed that four of the speakers, S4, S5, S8, and S10, did show a noticeable increase in SDST (and a further two, S2 and S7, a small increase), indicating less monotone speech as a result of treatment. The group results may have been affected by two speakers (S1 and S11) who showed noticeable decreases in SDST after treatment. These two speakers had pretreatment SDST values that were relatively high (ranking third and fourth in pretreatment SDST). These results underscore the advisability of considering qualitative analysis of individual speakers, in addition to statistical analysis of group results, in populations with speech disorder, which are notoriously heterogenous [24, 25].

 Tone was analysed perceptually using two different methods. The first employed transcription of isolated syllables extracted from the reading passage. Statistical analysis was not undertaken for this task, due to the low intrarater reliability. However, examination of the results indicated similar findings before and after therapy (mean accuracy of 54.02%, SD  =  0.16, before treatment and 56.62, SD  =  0.17, after treatment). Difficulties with transcribing tone from isolated syllables have been previously reported (e.g., Fok-Chan [26]). In view this, and of the poor intrarater reliability, a second task was employed: identification of error tones in phrases. This analysis revealed high tone accuracy before treatment (mean  =  97.72%, SD  =  0.03) and no significant difference in accuracy after treatment (mean  =  97.64%, SD  =  0.03; *P*  >  0.05). The finding of relatively intact lexical tone in Cantonese speakers with PD, as judged perceptually, was consistent with previous findings [8, 10]. 

 Acoustic analysis of tone also revealed no significant differences before and after treatment, for the group. This was consistent with the results our previous small-scale study [10]. However, examination of individual results showed that many of the participants (ten of the 12) showed flattened F0 configurations for target contour tones. This finding has been previously reported [10]. Improvement in some (but not all) contour tones was seen for three participants, after treatment. However, there was no change in the F0 patterns for the contour tones for the other participants who showed flattened patterns. Overall, the results of the acoustic analysis showed little to no improvement in lexical tones as a result of LSVT. 

 This study confirmed previous reports of relatively intact lexical tone in Cantonese speakers with Parkinson's disease [8, 10]. It is possible that this finding is related to the perceptual task involved. However, consistent results have been found in studies employing different perceptual methods. The finding of relatively intact lexical tone could also be related to the speech severity of the particular group of participants. However, in the current study, effort was made to recruit subjects with a wide range of speech severity. In addition, in the current study as well as our previous studies, the speakers with PD did have impairment of other speech dimensions associated with hypokinetic dysarthria (e.g., loudness, monotone, and speech rate). It seems that lexical tone may indeed be relatively preserved in this group of speakers. This may seem an anomaly, in a clinical population closely associated with disturbances at the laryngeal level [27]. However, it is consistent with reports of relatively intact tone in other Cantonese clinical populations (e.g., So and Dodd [28]; Stokes and Whitehill [29]). Several authors have attributed the relative robustness of tone in speech disordered populations to the high functional load of tone in Cantonese and other tone languages (e.g., So and Dodd [28]). However, this seems an unlikely explanation for individuals with PD, who have an acquired speech disorder as a result of neurological disease. The relative robustness of tone may be associated with the relatively small adjustments in FO needed for lexical contrasts, in contrast to FO fluctuations at the phrasal level for distinctions in intonation. Vance [9] hypothesized a possible differential control for tone and intonation, whereby lexical tone production might involve changes in laryngeal maneuvering while intonation might involve changes in subglottal pressure. However, there is no direct empirical evidence for this hypothesis. Dromey et al. [30] reported improvements in subglottal pressure and laryngeal control, following LSVT. Further studies are needed to explore the issue of possible differential control of these two speech components, both related to fine control of F0. Studies of speakers with hypokinetic dysarthria offer a unique contribution to this debate.
